# Genome wide association for substance dependence: convergent results from epidemiologic and research volunteer samples

**DOI:** 10.1186/1471-2350-9-113

**Published:** 2008-12-18

**Authors:** Catherine Johnson, Tomas Drgon, Qing-Rong Liu, Ping-Wu Zhang, Donna Walther, Chuan-Yun Li, James C Anthony, Yulan Ding, William W Eaton, George R Uhl

**Affiliations:** 1 Molecular Neurobiology Branch, NIH-IRP (NIDA), Suite 3510, 333 Cassell Drive Baltimore, Maryland 21224, USA; 2 Center for Bioinformatics, National Laboratory of Protein Engineering and Plant Genetic Engineering, College of Life Sciences, Peking University, Beijing, 100871, PR China; 3 Dept of Epidemiology, Michigan State University, East Lansing, MI 48824, USA; 4 Department of Mental Health and Hygiene, Johns Hopkins Bloomberg School of Public Health, Baltimore MD 21221, USA

## Abstract

**Background:**

Dependences on addictive substances are substantially-heritable complex disorders whose molecular genetic bases have been partially elucidated by studies that have largely focused on research volunteers, including those recruited in Baltimore. Maryland. Subjects recruited from the Baltimore site of the Epidemiological Catchment Area (ECA) study provide a potentially-useful comparison group for possible confounding features that might arise from selecting research volunteer samples of substance dependent and control individuals. We now report novel SNP (single nucleotide polymorphism) genome wide association (GWA) results for vulnerability to substance dependence in ECA participants, who were initially ascertained as members of a probability sample from Baltimore, and compare the results to those from ethnically-matched Baltimore research volunteers.

**Results:**

We identify substantial overlap between the home address zip codes reported by members of these two samples. We find overlapping clusters of SNPs whose allele frequencies differ with nominal significance between substance dependent *vs *control individuals in both samples. These overlapping clusters of nominally-positive SNPs identify 172 genes in ways that are never found by chance in Monte Carlo simulation studies. Comparison with data from human expressed sequence tags suggests that these genes are expressed in brain, especially in hippocampus and amygdala, to extents that are greater than chance.

**Conclusion:**

The convergent results from these probability sample and research volunteer sample datasets support prior genome wide association results. They fail to support the idea that large portions of the molecular genetic results for vulnerability to substance dependence derive from factors that are limited to research volunteers.

## Background

Vulnerability to substance dependence is a complex trait with strong genetic influences that are well documented by data from family, adoption and twin studies [1-4]. Twin studies support the view that much of the heritable influence on vulnerability to dependence on addictive substances from different pharmacological classes (*eg *nicotine and stimulants) is shared [2,3,5]. Combined data from linkage and genome wide association (GWA) datasets [6-11] suggest that most of the genetics of vulnerability to dependence on addictive substances is likely to be polygenic, arising from variants in genes whose influences on vulnerability, taken one at a time, are relatively modest. Substance-dependent individuals also differ from control individuals in personality, cognitive domains and co-occurrence of psychiatric diagnoses [1,12] (reviewed in [13]).

GWA approaches of increasing sophistication have been developed and used to identify the specific genes and genomic variants that predispose to vulnerability to substance dependence. For example, we have assembled a group of research volunteers from the Molecular Neurobiology Branch of the NIH (NIDA) intramural research program in Baltimore between 1990 and 2008 ("MNB"). We have compared allele frequencies at *ca *1500, 10,000, 100,000, 500,000 and then 1,000,000 SNP markers in increasing numbers of substance dependent *vs *control individuals from this growing sample, including 680 substance dependent or control individuals with self reported European ancestries [6-9,11], (Drgon et al, submitted).

There is the theoretical concern that this MNB sample, and many of the other samples collected for studies of genetics of dependence on addictive substances, might be biased based on the requirement that subjects were ascertained when they volunteered for research. It is conceivable that "volunteering" might interact with heritable features of personality, cognitive, psychiatric and/or other features by which substance dependent individuals might differ from controls [1,12-25]. GWA findings in such research volunteer samples would then conceivably provide a distorted representation of findings that would otherwise be made in members of the community.

The Baltimore site of the Epidemiological Catchment Area (ECA) Study provides a good comparison group to probe such potential confounding features [24,26]. This study initially assembled a probability sample of individuals who represented the East Baltimore population, including many of the census tracts in which MNB research volunteers reported their home residences. ECA investigators followed substantial portions of these individuals, interviewing them four times and sampling DNA from most of the 1071 individuals from the initial sample who were interviewed in 2004–05 (*see below*). The repeated assessment of these individuals provides confident assessment of dependence-related phenotypes that include DSM diagnoses of substance abuse and dependence and Fagerstrom Test for Nicotine Dependence (FTND) diagnoses of nicotine dependence.

We thus now report data that confirms the overlapping areas of Baltimore from which ECA and MNB subjects were sampled. We report genome wide association studies for substance dependence phenotypes for Baltimore ECA subjects. We compare these genome wide association results with those from ethnically-matched MNB research volunteers who were recruited from many of the same areas. We discuss the significance of the substantially-overlapping data that we report, as well as the limitations of the samples and datasets. These data document large molecular genetic overlaps between probability-sample and research volunteer samples for substance dependence.

## Methods

### ECA Sample

Subjects from the Baltimore (Eastern Baltimore Mental Health Survey) site for the Epidemiological Catchment Area Program (ECA) were ascertained as a probability sample of individuals in dwelling units within census tracts near the Johns Hopkins Medical Institutions and initially interviewed in 1981 [24,26]. Subsets of these individuals were interviewed in 1982, 1993–96 and 2004–5. Diagnoses came from the Diagnostic Interview Schedule (DIS), a self-report instrument [27] whose validity and reliability has been documented in this sample [27a]. Smoking was also described using the Fagerstrom Test for Nicotine Dependence (FTND) [28-30]. Although many of the individuals who were > 65 in 1981 had died by the 2004–05 follow-up, self-report survey data was collected from 662 European-American respondents (63% female) during this follow-up. This subset provided good representation of the composition of the portion of the original Baltimore ECA cohort that was of this race/ethnicity. Blood for DNA extraction and for lymphocyte immortalization was obtained from 74% of these individuals, who did not differ from subjects from whom DNA was not obtained in any obvious feature that related to substance dependence [30a].

Individuals who were dependent on an abused substance were identified by DSM criteria, except for nicotine dependence which was based on FTND criteria. Eighty substance dependent individuals were identified. These individuals were matched for gender and age to eighty control individuals. These control individuals were East Baltimore ECA participants who never used any illegal drugs more then 5 times in their lives, were not dependent on any drug or alcohol, drank less than one drink per day (waves 3 and 4) drank less than 5 drinks/week (waves 1 and 2) and had FTND scores < 7. Generation and analyses of these data were approved by the Johns Hopkins Bloomberg School of Public Health IRB and exempt protocols for pooled genotyping approved by the NIH Office of Human Research Subject Protection.

### Comparison research volunteer sample

Data from these European-American ECA subjects was compared to data from European-American MNB research volunteers who provided informed consents, ethnicity data, drug use histories and DSMIII-R or IV diagnoses as previously described [6,31,32]. DNA in 34 pools sampled 400 "abusers" with heavy lifetime use of illegal substances and, for virtually all, DSMIII-R/IV dependence on at least one illegal abused substance and 280 "controls" who reported no significant lifetime use of any addictive substance. Generation and analyses of these data were approved by the NIH IRP (NIDA) IRB (protocol #148) and exempt protocols for pooled genotyping approved by the NIH Office of Human Research Subject Protection.

### DNA preparation and assessment of allelic frequencies

DNA was prepared from blood (MNB and some ECA subjects) or cell lines (most ECA subjects) [6,31,32] and carefully quantitated. DNAs from groups of 20 individuals of the same phenotype were combined. Hybridization probes were prepared with precautions to avoid contamination, as described (Affymetrix assays 500 k [9,11,33,34]). 150 ng of pooled DNA was digested using *Sty*I or *Nsp*I, ligated to appropriate adaptors and amplified using a GeneAmp PCR System 9700 (Applied Biosystems, Foster City, CA) with 3 min 94°C, 30 cycles of 30 sec 94°C, 45 sec 60°C, 15 sec at 68°C and a final 7 min 68°C extension. PCR products were purified (MinEluteTM 96 UF kits, Qiagen, Valencia, CA) and quantitated. Forty μg of PCR product was digested for 35 min at 37°C with 0.04 unit/μl DNase I to produce 30–100 bp fragments which were end-labeled using terminal deoxynucleotidyl transferase and biotinylated dideoxynucleotides and hybridized to the appropriate 500 k array (*Sty I *or *Nsp I *arrays) (Mendel array sets, Affymetrix). Arrays were stained and washed as described (Affymetrix Genechip Mapping Assay Manual) using immunopure strepavidin (Pierce, Milwaukee, WI), biotinylated antistreptavidin antibody (Vector Labs, Burlingame, CA) and R-phycoerythrin strepavidin (Molecular Probes, Eugene, OR). Arrays were scanned and fluorescence intensities quantitated using an Affymetrix array scanner as described [9,11,33,34].

Chromosomal positions for each SNP were sought using NCBI (Build 36.1) and NETAFFYX (Affymetrix) data. Allele frequencies for each SNP in each DNA pool were assessed based on hybridization intensity signals from four arrays, allowing assessment of hybridization to the 12 "perfect match" cells on each array that are complementary to the PCR products from alleles "A" and "B" for each diallelic SNP on sense and antisense strands. We eliminated: i) SNPs on sex chromosomes and iii) SNPs whose chromosomal positions could not be adequately determined.

Each array was analyzed as described [9,11,33,34], with background values subtracted, normalization to the highest values noted on the array, averaging of the hybridization intensities from the array cells that corresponded to the perfect match "A" and "B" cells, calculation of "A/B ratios" by dividing average normalized A values by average normalized B values, arctangent transformations to aid combination of data from arrays hybridized and scanned on different days, and determination of the average arctangent value for each SNP from the 4 replicate arrays. A "t" statistic for the differences between abusers and controls was generated as described [9,11,33,34] for each SNP. We focused on SNPs that displayed t statistics with p < 0.05 for abuser/control differences. We sought evidence for clustering of these SNPs by focusing on chromosomal regions in which at least three of these outlier SNPs, assessed by at least two array types, lay within 25 Kb of each other. We term these clustered, nominally-positive SNPs "clustered positive SNPs", and focus our analyses on regions in which they lie.

To confirm the SNPs within the positive clusters from the current dataset, we sought convergence between data from these clustered nominally-positive SNPs and clustered nominally-positive SNPs, determined in the same way, from 1 M SNP genome wide association studies of the MNB samples (Drgon et al, submitted). To provide insights into some of the genes likely to harbor variants that contribute to individual differences in vulnerability to substance dependence, we sought candidate genes that were identified by overlapping clusters of positive SNPs from each of these samples.

We compare observed results to those expected by chance using Monte Carlo simulation trials, as described [9,11,33,34]. For each trial, a randomly-selected set of SNPs from the current dataset was assessed to see if it provided results equal to or greater than the results that we actually observed. The number of trials for which the randomly-selected SNPs displayed (at least) the same features displayed by the observed results was then tallied to generate an empirical p value. These simulations thus correct for the number of repeated comparisons made in these analyses, an important consideration in evaluating these GWA datasets. We thus focus on genes which display convergence between nominally-significant results obtained from the two dependence *vs *control samples. We report Monte Carlo probabilities for the observed convergence of clustered nominally positive SNPs within each gene, using simulations that correct for the number of repeated comparisons.

To assess the power of our current approach, we use current sample sizes and standard deviations, the program PS v2.1.31 [35,36] and α = 0.05. To provide controls for the possibilities that abuser-control differences observed herein were due to a) occult ethnic/racial allele frequency differences or b) noisy assays, we assessed the overlap between the results obtained here and the SNPs that displayed the largest a) allele frequency differences between African-American *vs *European-American control individuals and b) the largest assay "noise".

We have compared the patterns of human brain expression for the genes identified herein to those identified using a novel tool based on the distribution of expressed sequence tags (ESTs) contained in an annotated set of brain cDNA libraries (CYL, GRU et al, *in preparation*). Briefly, we identified 846 human cDNA libraries with cDNAs represented in dbEST. These libraries were constructed from regions of brains that appeared to display modest or no pathology. We based the analyses on two sets of criteria: 1) all entries in these libraries and 2) "more reliable" entries that display i) correct genomic orientation and either ii a) evidence for polyA tail or ii b) spliced structure (CYL, GRU et al, *in preparation*). For each brain region, we assessed the *p*-value for over-representation of expression of the dependence-associated genes using hypergeometric distribution tests with false discovery rate (FDR) corrections. *Q*-values < 0.05 were considered statistically significant.

## Results

**Figure 1:**
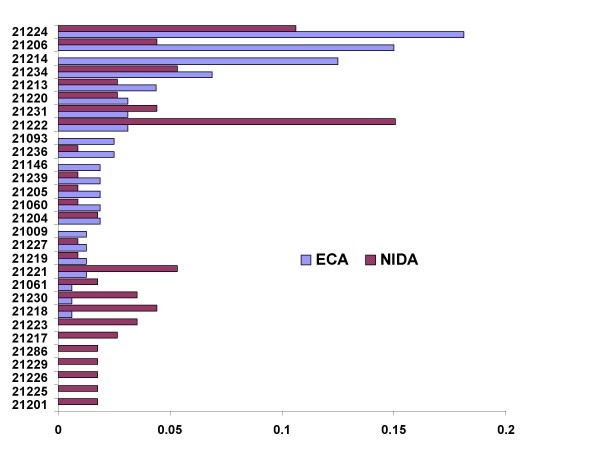
**Substantial overlaps of the zip codes in which subjects reported their residences.** Fractional distributions (*X axis*) of zip codes (*Y axis*) in which ECA (*solid blue bars*) or MNB (*dotted purple bars*) subjects reported their residence. Areas from which NIDA and ECA recruitment efforts were dissimilar include 21214; Lauraville and surrounding areas and 21222, Dundalk and surrounding areas. Zip codes from which fewer than 3 individuals were recruited are not indicated.

We assessed the extent to which the ECA and NIDA research volunteers came from similar Baltimore neighborhoods by comparing the distributions of available zip codes from these samples. As noted in Figure 1, there was substantial overlap between these zip code distributions, providing impetus for the comparative molecular genetic studies described below.

We then assessed allele frequencies in multiple pools of DNA from substance dependent and control ECA individuals. There was modest variability among replicate arrays that assessed the same pool (standard error of the mean (SEM) 0.032) and among the different pools that assessed the same phenotype (SEM 0.033). These samples and these estimates of variance thus provided 0.9, 0.74, 0.46 and 0.21 power to detect allele frequency differences of 12.5, 10, 7.5 and 5%, respectively.

28,137 SNPs displayed "nominally positive" t values with p < 0.05 in these ECA samples. 7,620 of these nominally positive SNPs fell into 1660 clusters of at least 3 SNPs that came from both array types were separated from adjacent nominally-positive SNPs by less than 25,000 basepairs. Monte Carlo simulations reveal p < 0.00001 for this degree of clustering.

One hundred seventy two genes are identified by both 1) clusters of nominally-positive SNPs from the ECA samples and 2) overlapping clusters of nominally positive SNPs from MNB samples. We list the 126 of these genes whose nominal Monte Carlo p values are < 0.05 in Table 1. This number of genes is never identified by chance in both samples by any of 10,000 Monte Carlo simulation trials (thus, p < 0.0001). There is also overlap, to extents greater than expected by chance, with the clusters of SNPs whose allele frequencies distinguish MNB African-American polysubstance abusers from controls (p < 0.0001) [9], Japanese methamphetamine abusers from controls (p < 0.0001) [11], Taiwanese methamphetamine abusers from controls (p < 0.0007) [11] and more-frequently nicotine dependent smokers of European ancestry from less-frequently nicotine dependent smokers (p < 0.0001) [10].

**Table 1:** Genes that contain overlapping clusters of nominally positive SNPs in both ECA and European-American MNB research volunteer samples that display nominal p < 0.05.

				***Clust SNPs***		
***gene***	***ch***	***bp***	***description***	***ECA***	***MNB***	***p***
A2BP1	16	6,009,133	ataxin 2-binding protein 1	16	42	0.0038
ACCN1	17	28,364,218	neuronal amiloride-sens cation chan 1	5	5	0.0240
ADARB2	10	1,218,073	RNA spec A deaminase B2	4	8	0.0420
ADCY2	5	7,449,345	adenylate cyclase 2	7	6	0.0210
AGBL4	1	48,822,129	ATP/GTP binding protein-like 4	3	8	0.0400
AK5	1	77,520,330	adenylate kinase 5	10	4	0.0090
AKAP6	14	31,868,274	A kinase anchoring protein 6	3	15	0.0210
ALK	2	29,269,144	anaplastic lymphoma kinase (Ki-1)	6	10	0.0470
ANKFN1	17	51,585,835	ankyrin-rep Fn III dom cont 1	3	14	0.0130
ATXN1	6	16,407,322	ataxin 1	3	17	0.0120
C18orf1	18	13,208,795	chromosome 18 open reading frame 1	4	6	0.0440
C3orf21	3	196,270,302	chromosome 3 open reading frame 21	4	4	0.0380
C8A	1	57,093,065	complement component 8 α polypep	3	7	0.0180
C9orf88	9	129,307,439	Chr 9 open reading frame 88	4	4	0.0220
CABIN1	22	22,737,765	calcineurin binding protein 1	4	4	0.0220
CACNA2D3	3	54,131,733	voltage det Ca chan α2/δ3 subunit	12	18	0.0091
CCBE1	18	55,252,124	collagen and calcium binding EGF domains 1	7	10	0.0090
CCDC63	12	109,769,194	coiled-coil domain containing 63	3	4	0.0290
CD180	5	66,513,872	CD180 molecule	5	5	0.0051
CDH13	16	81,218,079	cadherin 13	18	65	0.0019
CDH23	10	72,826,697	cadherin-like 23	7	4	0.0380
CGNL1	15	55,455,997	cingulin-like 1	3	11	0.0040
CHL1	3	213,650	close homolog of L1	3	12	0.0103
CHST11	12	103,370,614	chondroitin 4 sulfotransferase 11	4	4	0.0470
CIT	12	118,607,981	citron rho-interacting, serine/threonine kinase 21	5	6	0.0160
CNTN5	11	98,397,081	contactin 5	11	10	0.0390
CNTNAP2	7	145,444,386	contactin associated protein-like 2	22	9	0.0380
CPVL	7	29,001,772	carboxypeptidase vitellogenic-like	4	4	0.0290
CRYL1	13	19,875,810	crystalline λ1	4	5	0.0170
CSMD1	8	2,782,789	CUB and Sushi multiple domains 1	29	137	0.0014
CUGBP2	10	11,087,290	CUG triplet repeat RNA bind prot 2	16	4	0.0031
DAB1	1	57,236,167	disabled homolog 1	4	24	0.0140
DLC1	8	12,985,243	deleted in liver cancer 1	13	9	0.0059
DNAPTP6	2	200,879,041	DNA polymerase-transactivated protein 6	8	4	0.0140
DOCK2	5	168,996,871	dedicator of cytokinesis 2	3	7	0.0490
DPP6	7	154,060,464	dipeptidyl-peptidase 6	6	4	0.0250
EDNRA	4	148,621,575	endothelin receptor type A	3	4	0.0280
EFCAB4B	12	3,627,370	EF-hand calcium binding domain 4B	3	6	0.0165
EPHB1	3	135,996,950	EPH receptor B1	13	5	0.0090
ESRRG	1	214,743,211	estrogen-related receptor γ	3	14	0.0210
EVI1	3	170,285,244	ecotropic viral integration site 1	3	12	0.0047
F5	1	167,750,033	coagulation factor V	4	11	0.0049
FAM13A1	4	89,866,129	family with seq sim 13 A1	6	4	0.0360
FAM3C	7	120,776,141	family with sequence similarity 3 C	6	4	0.0109
FAM3D	3	58,594,710	family with sequence similarity 3 D	7	4	0.0063
FBXL17	5	107,223,348	F-box and leucine-rich repeat protein 17	6	6	0.0430
FGD2	6	37,081,401	FYVE, RhoGEF PH dom cont 2	3	4	0.0320
FHIT	3	59,710,076	fragile histidine triad gene	24	62	0.0030
FLJ11151	16	12,664,438	hypothetical protein FLJ11151	4	4	0.0380
FLJ32682	13	45,013,433	hypothetical protein FLJ32682	4	5	0.0180
FN1	2	215,933,422	fibronectin 1	3	5	0.0260
FOXP1	3	71,087,426	forkhead box P1	4	17	0.0180
FREM3	4	144,717,905	FRAS1 related extracellular matrix 3	4	6	0.0130
FRMD4A	10	13,725,718	FERM domain containing 4A	3	23	0.0090
GABBR2	9	100,090,187	GABA B receptor 2	11	6	0.0073
GLIS3	9	3,817,676	GLIS family zinc finger 3	13	18	0.0016
GRB10	7	50,625,259	growth factor receptor-bound protein 10	3	13	0.0083
GRID1	10	87,349,292	delta 1 inotropic glutamate rec	7	18	0.0130
GRIK1	21	29,831,125	kainate 1 inotropic glutamate rec	4	12	0.0150
GTF2F2L	4	148,646,691	general transcription fact IIFpolypep 2-L	3	3	0.0170
HPSE2	10	100,208,867	heparanase 2	6	18	0.0160
HS3ST4	16	25,611,240	heparan sulfate 3-O-sulfotransferase 4	4	11	0.0250
IMPA2	18	11,971,455	inositol(myo)-1(or 4)-monophosphatase 2	6	6	0.0064
IQGAP2	5	75,734,905	IQ motif cont GTPase activ prot 2	7	5	0.0170
JAKMIP1	4	6,106,385	janus kinase microtubule interacting protein 1	3	8	0.0138
KCNB1	20	47,421,912	Shab-rel volt-gated K chan 1	5	3	0.0190
KCNIP4	4	20,339,337	Kv channel interacting protein 4	13	9	0.0107
KCNJ6	21	37,918,655	inwardly-rect K chan J 6	3	8	0.0330
KCNMA1	10	78,299,366	large conduct Ca-act K chan M α1	8	8	0.0390
KIAA1576	16	76,379,984	KIAA1576 protein	3	6	0.0260
KREMEN1	22	27,799,106	kringle cont TM prot 1	5	4	0.0190
KSR2	12	116,389,387	kinase suppressor of ras 2	5	4	0.0430
LDLRAD3	11	35,922,188	low density lipoprotein recep cl A dom cont 3	3	6	0.0330
LTF	3	46,452,500	lactotransferrin	3	4	0.0290
MAGI1	3	65,314,946	membr-assoc G kinase WW PDZ dom cont 1	6	9	0.0380
MAGI2	7	77,484,310	membrane assoc G kinase WW PDZ dom 2	9	16	0.0430
MGC23985	5	147,252,464	similar to AVLV472	3	7	0.0085
MICAL2	11	12,088,714	calponin LIM cont microtub monoxygenase 2	8	8	0.0120
MTSS1	8	125,632,212	metastasis suppressor 1	9	9	0.0050
MTUS1	8	17,545,583	mitochondrial tumor suppressor 1	3	4	0.0090
MYO18B	22	24,468,120	myosin XVIIIB	15	8	0.0460
MYO3A	10	26,263,202	myosin IIIA	3	5	0.0018
NAALADL2	3	176,059,805	N-Ac α-linked acidic dipeptidase-L 2	4	5	0.0340
NFIA	1	61,320,881	nuclear factor I/A	4	4	0.0080
NLGN1	3	174,598,938	neuroligin 1	4	4	0.0260
OPCML	11	131,790,085	opioid binding protein/cell adhesion molecule-L	10	13	0.0076
PALM2	9	111,442,893	paralemmin 2	10	16	0.0220
PALM2-AKAP2	9	111,582,410	PALM2-AKAP2 protein	5	10	0.0021
PARD3B	2	205,118,761	par-3 partitioning defective 3 homolog B	3	5	0.0160
PDE4D	5	58,302,468	cAMP spec phosphodiesterase 4D	4	4	0.0140
PKD1L2	16	79,691,991	polycystic kidney disease 1-like 2	4	4	0.0060
PLD5	1	240,318,895	phospholipase D family 5	3	16	0.0300
PRKCA	17	61,729,388	protein kinase C α	6	4	0.0113
PRKCH	14	60,858,268	protein kinase C η	7	22	0.0300
PRKG1	10	52,504,299	cGMP dep protein kinase I	12	10	0.0014
PRPF4	9	115,077,795	PRP4 pre-mRNA process fact 4 homol	6	4	0.0360
PSD3	8	18,432,343	pleckstrin and Sec7 domain containing 3	8	16	0.0240
PTPN14	1	212,597,634	no rec prot Y phosphatase 14	10	4	0.0150
PTPRK	6	128,331,625	recept protein tyrosine phosphatase K	3	8	0.0062
PTPRT	20	40,134,806	recept prot Y phosphatase T	3	15	0.0190
RBMS3	3	29,297,947	sing strand RNA binding motif interact prot	5	13	0.0230
ROR2	9	93,524,705	receptor tyrosine kinase-L orphan recept 2	5	4	0.0290
RORA	15	58,576,755	RAR-related orphan receptor A	4	9	0.0220
SLC2A13	12	38,435,090	solute carrier family 2 13	4	4	0.0086
SLIT3	5	168,025,857	slit homolog 3	3	4	0.0370
SRGAP3	3	8,997,278	SLIT-ROBO Rho GTPase activating protein 3	4	23	0.0087
STK32B	4	5,104,428	serine threonine kinase 32B	3	25	0.0011
STK39	2	168,518,777	serine threonine kinase 39	4	4	0.0036
SYNE1	6	152,484,516	spectrin rep cont nuclear envelope 1	7	5	0.0034
TACC2	10	123,738,679	transforming acidic coil-coil cont prot 2	3	9	0.0430
TBC1D22A	22	45,537,213	TBC1 domain family 22A	3	8	0.0270
TEK	9	27,099,286	TEK tyrosine kinase, endothelial	3	9	0.0420
TG	8	133,948,387	thyroglobulin	4	4	0.0180
THSD4	15	69,220,842	thrombospondin I dom cont 4	3	11	0.0063
TMEM132C	12	127,318,855	transmembrane protein 132C	5	13	0.0084
TMEM132D	12	128,122,224	transmembrane protein 132D	8	11	0.0090
TMEM16D	12	99,712,716	transmembrane protein 16D	11	11	0.0320
TMTC1	12	29,545,024	transmemb tetratricopep rep cont 1	3	3	0.0035
TRPC4	13	37,108,795	transient receptor potential cation channel C 4	8	4	0.0140
TULP4	6	158,653,680	tubby like protein 4	6	9	0.0076
UNC5C	4	96,308,712	unc-5 homolog C	9	4	0.0162
VAMP4	1	169,938,783	vesicle-associated membrane protein 4	3	5	0.0170
VAPB	20	56,397,651	vesicle-assoc memb protein-assoc prot B C	4	11	0.0034
VIT	2	36,777,418	Vitrin	3	9	0.0110
ZNF365	10	63,803,957	zinc finger protein 365	6	4	0.0340
ZNF406	8	135,559,213	zinc finger protein 406	16	4	0.0014

The numbers of nominally-positive SNPs that lay in clusters within the gene's exons and in 10 kb genomic flanking regions are noted for each sample. Chromosome number and initial chromosomal position for the cluster (bp, NCBI Mapviewer Build 36.1) are listed. Nominal p values for each gene are based on 10,000 Monte Carlo simulation trials. For each trial, the number of times randomly-selected segments of the genome that lie within genes are assessed for the same features displayed by the actual gene identified. Note that the very highly significant p values for the overall convergence noted between these two datasets (text) does account for multiple comparisons, while the much more modest p values for many of the individual genes (displayed here) do not. Genes that are identified by clustered nominally positive SNPs in both samples but whose gene-wise p values lie > 0.05 (perhaps, in part, due to the large size of the genes) include: C4orf13, PRKCE, UNC13C, KIAA1303, MYR8, DNAH11, ONECUT2, TGFBR3, TPD52L1, C9orf28, TMEM108, GALNT14, HECW2, NFIB, STS-1, KIAA1217, RAB3C, SNTG1, CPNE4, PTPRM, SLC39A11, MDS1, GPC5, ZNF533, NR5A2, RYR3, C8orf68, CTNNA2, GRM5, ATRNL1, ARL15, BTBD9, CNTNAP5, GALNTL4, PELI2, SNRPN, GPR98, ERC2, NFATC2, FLJ16124, GRM7, SORCS2, NPAS3, PARVB, IGL@ and LRP1B.

We would anticipate the observed, highly-significant clustering of SNPs that display nominally-positive results if many of these reproducibly-positive SNPs lay near and were in linkage disequilibrium with functional allelic variants that distinguished substance-dependent subjects from control subjects. We would not anticipate this degree of clustering if the results were solely due to chance. The Monte Carlo p values noted here are thus likely to receive contributions from both the extent of linkage disequilibrium among the clustered, nominally-positive SNPs and the extent of linkage disequilibrium between these SNPs and the functional haplotype(s) that lead to the association with substance dependence.

Neither controls for occult stratification nor for assay variability appear to provide convincing alternative explanations for most of the data obtained here. When we examined the overlap between the 7620 clustered positive SNPs from the ECA samples and: 1) the 2.5% of the SNPs for which the noise in validating studies was highest and 2) the 2.5% of SNPs that displayed the largest differences between Baltimore African-American *vs *European American control individuals, we found 15 and 245, respectively, *vs *185 expected by chance in each case.

We evaluated evidence for preferential brain and brain regional expression of the 172 genes identified in Table 1 (body and legend). Brain libraries represented in dbEST contained ESTs that corresponded to 91% (157/172) of these genes. Expression for this set of genes (*compared to all genes*) displayed nominal significance in thalamus (p < 10^-8^), amygdale (p = 5.6 × 10^-9^), hippocampus (p = 2.6 × 10^-5^), frontal cortex (p = 0.015) and medulla (p = 0.039) using hypergeometric tests (p values were Bonferroni corrected for repeated comparisons). Assessments of the "more reliable" subset of ESTs revealed significant overexpression in whole brain (p = 2.1 × 10^-7^), amygdala (p = 4.4 × 10^-6^), hippocampus (p = 0.0011), cerebellum (p = 0.0052), thalamus (p = 0.037) and cortex (p = 0.049) (p-values were Bonferroni corrected).

## Discussion

The current results 1) provide independent support for GWA results from larger samples of research volunteers studied for substance dependence phenotypes and 2) provide a control for one of the potential confounding features of this previously-studied sample. The possibility that genetic results from members of any sample of research volunteers might not represent the genetics of members of the general population is ever present. In molecular genetic studies of substance dependence, however, features that are both 1) heritable and 2) differentially present in substance dependent individuals might, *a priori*, be considered to be especially likely to provide confounding influences. Cognitive abilities are highly heritable. Cognitive tests in a number of samples of substance dependent individuals have indicated differences in performance (reviewed in [13]). A study in twin pairs whose members were discordant for substance use concluded that most of these cognitive differences were likely to be heritable antecedents to, not just consequences of, the use of addictive substances [37]. Cognitive abilities have been shown to interact with willingness to volunteer for and/or participate in research protocols in a number of settings [1,12,14-25]. A number of personality features are also highly heritable [38]. Neuroticism is both one of the more heritable personality features and also the personality feature that has been demonstrated to be elevated in several samples of substance dependent individuals [38]. Personality features have also been linked to willingness to volunteer for participation in research protocols [1,12,14-25]. A number of psychiatric disorders that might also be linked to differential willingness (or ability) to participate as a research volunteer are also heritable and co-occur with substance dependences at rates much greater than chance [13].

It is also important to keep a number of limitations in mind in considering the present results. 1) The preplanned approach used here demands that multiple nominally-positive SNPs from each sample tag the same genomic region that lies within a gene. Requirements that nominally positive SNPs from the current dataset come from each of the two 500 k array types add a technical control. Monte Carlo approaches that do not require specification of underlying distributions can readily judge the degree to which all of the observations made here could be due to chance. Nevertheless, there have been no unanimous criteria for declaring "replication" or "convergence" for GWA studies, a consideration worth considering in evaluating the current results. 2) The ECA samples are of modest size, limited by the numbers of substance abusing or dependent individuals in the aging Baltimore ECA cohort follow-up samples. Power calculations that document the modest power in European-Americans samples revealed even more modest power for the smaller number of African-American substance dependent individuals in this sample; we have thus not analyzed these samples. Modest power limits interpretation of negative data, substantially restricting inferences about genes identified in the more robust dataset from MNB research volunteers but not in these ECA samples. 3) There is very highly significant confidence in the overall set of convergent positive results reported here. However, the values for each gene, tested individually, provide much more modest levels of statistical assurance. 4) Focus on data from autosomes here allows us to combine data from male and female subjects, but misses potentially important contributions from sex chromosomes. 5) The individuals in the Baltimore ECA cohort were not initially sampled based on their willingness to be volunteers. However, participants needed to consent in order to be able to be followed and studied genetically. Although the overwhelming majority of the European-American participants who were followed did consent to participation in genetic studies, potential contributions that the non-consenting individuals might have made to the present results remain unknown. 6) The pooling approach that we use here provides excellent correlations between individually-genotyped and pooled allele frequency assessments in validation experiments. This approach has allowed us to use these samples without adding additional confidentiality burdens to these intensively-studied individuals. Nevertheless, estimates of allele frequencies based on pooled data represent approximations of "true" allele frequency differences that might be determined by error free individual genotyping of each participant. 7) There is no indication that the overall positive results reported here are based on the SNPs whose assays provide more noise, and no indication that occult stratification on racial/ethnic lines contributed overall to the results that we obtain here. However, we cannot totally exclude contributions of occult stratification that cannot be detected by these overall screens to findings in specific genes. 8) The convergent data derived from studies of individuals with dependence on substances in several different pharmacological classes supports the idea that many allelic variants enhance vulnerability to dependence on a number of substances. These results do not exclude additional contributions from genomic variants that influence vulnerability to specific substances. 9) We focus on identification of genes. Although associations away from annotated genes can also provide interesting results, the genes that we identify in the present work provide a number of interesting views of substance dependence. These data reinforce our observations that many of these genes are likely to contribute to brain differences that are reflected in the mnemonic aspects of addiction, and that some of them also provide tempting targets for antiaddiction therapeutics. We discuss these ideas in more detail elsewhere [12,13].

More of the genes identified here are represented among cDNAs cloned from brain libraries than is the case for all human genes. The results focus attention on expression in hippocampus, which manifests interesting roles in mnemonic processes and cerebral cortical connections that may provide additional clues to the pathophysiology of human substance dependence. Although detailed discussion of each of these groups of genes is beyond the scope of this report, it is interesting to note that about 15% of the genes enumerated in Table 1 can be related to cell adhesion mechanisms. This is a much larger fraction that the fraction of all genes, about 2%, that are identified as cell adhesion molecules in a recent bioinformatic approach to comprehensively identifying cell adhesion molecules [39], supporting overrepresentation of these genes among addiction-associated genes.

A number of the genes identified in this work are also identified in genome wide association and/or candidate gene datasets for heritable disorders or phenotypes that co-occur with addictions. As we discuss elsewhere, dependence-vulnerability GWA results overlap at levels greater than expected by chance with GWA studies of cognitive abilities, personality features, frontal lobe brain volumes and bipolar disorder [13].

## Conclusion

The observations in the present dataset that the findings from a population-based sample converge strongly with those made in larger research volunteer samples are reassuring. They support the idea that many of the molecular genetic findings that we and others have previously reported are not due simply to the methods used for ascertainment of "cases" and "controls" for our studies in research volunteers. It is important to note that this overall conclusion does not exclude contributions for some of these sampling issues to findings in particular genes. Nevertheless, the findings presented here promise to add to ongoing processes for comparing GWA datasets from research volunteers to those from population based samples. For dependence on alcohol, tobacco and other drugs, as for many complex disorders, such data provides an increasingly rich basis for improved understanding and for personalization of prevention and treatment strategies.

## Competing interests

The authors declare that they have no competing interests.

## Abbreviations

DSM: diagnostic and statistical manual; ECA: Epidemiological catchment area; MNB: Molecular Neurobiology Research Branch.

## Authors' contributions

CJ performed data manipulation, analysis and participated in the interpretation of the results. TD participated in DNA pool preparation, data acquisition, data analysis and manuscript preparation. QRL and PWZ participated in probe synthesis and data acquisition. DW performed the DNA and DNA pool preparation and participated in data acquisition. CYL participated in data analysis. JA participated in conception of the study and worked with the ECA study. YD helped to identify participants with specific clinical characteristics. WE participated in the conception of the study, study design and funding, and interpretation of the results. GRU conceived the study, participated in study design, data analysis and drafted and edited the manuscript. All authors read and approved the final manuscript.

## Pre-publication history

The pre-publication history for this paper can be accessed here:


